# Pediatric Pleomorphic Adenoma of the Palate

**DOI:** 10.1155/2021/9938672

**Published:** 2021-05-17

**Authors:** Jerome A. Lindeboom, Jean-Pierre T. F. Ho, Naomi Donner, Willem H. Schreuder

**Affiliations:** ^1^Department of Oral and Maxillofacial Surgery, Amsterdam University Medical Centers and Amstelland Hospital, University of Amsterdam, Netherlands; ^2^Department of Oral and Maxillofacial Surgery, Amsterdam University Medical Centers and Northwest Clinics, University of Amsterdam, Netherlands; ^3^Department of Pathology, Amsterdam University Medical Centers, University of Amsterdam, Amsterdam, Netherlands; ^4^Department of Oral and Maxillofacial Surgery, Amsterdam University Medical Centers, University of Amsterdam, Amsterdam and Department of Head and Neck Surgery, Antoni van Leeuwenhoek Hospital, Amsterdam, Netherlands

## Abstract

Pleomorphic adenoma is the most common salivary gland tumor but is extremely rare in pediatric patients. The parotid gland is the most affected salivary gland, and the minor salivary glands are rarely affected. Here, we report a case of a 12-year-old boy with a pleomorphic adenoma of the palate.

## 1. Introduction

Salivary tumors are extremely rare in children, with fewer than 5% occurring in children compared to adults. Tumors in the minor salivary glands are uncommon, and pleomorphic adenomas are the most common benign tumors of the palate. Pleomorphic adenoma has a remarkable degree of morphological diversity, with the essential components being the capsule, epithelial and myoepithelial cells, and the mesenchymal or stromal elements [1].

The tumor consists of acini, cords, and thin strands of epithelial cells suspended in stroma, which often has a myxomatous appearance [2]. The tumor is predominately seen in the parotid gland. On the palate, the tumor is mostly located at the transition of the hard and soft palate [3]. Pediatric pleomorphic adenoma of the palate is slightly more common in girls than boys (1.3 : 1), but due to the rarity, it is difficult to provide a reliable estimate [2].

## 2. Case Report

**Figure 1:**
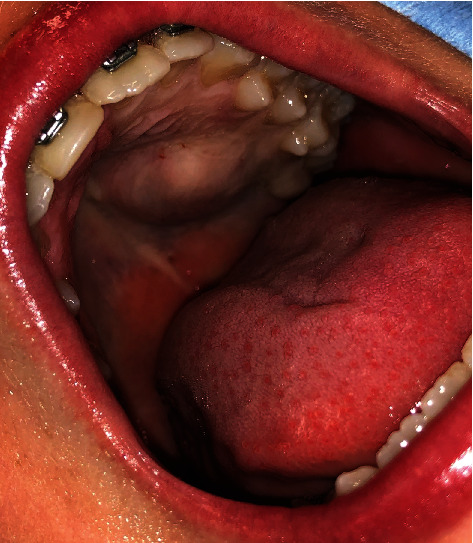
Photograph of the swelling of the left palate with intact overlying mucosa.

**Figure 2:**
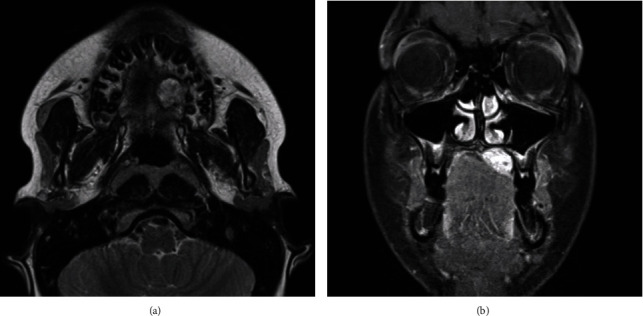
MRI sections showing a well-defined, homogenous enhancing lesion in the left palate.

**Figure 3:**
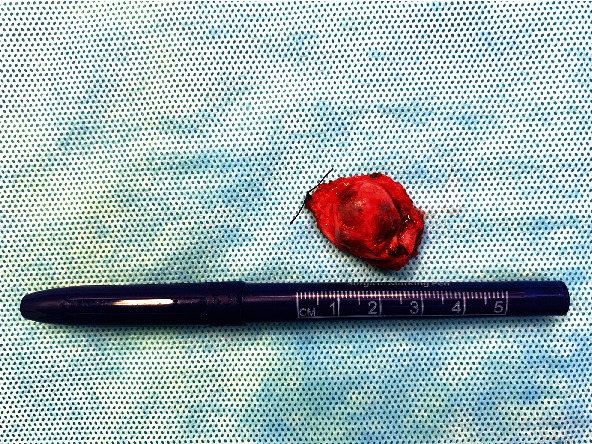
Image of the excised palatal tumor, with macroscopically intact capsule.

**Figure 4:**
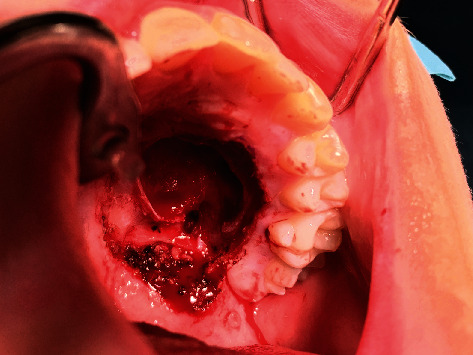
Image of the palatal defect after tumor resection.

**Figure 5:**
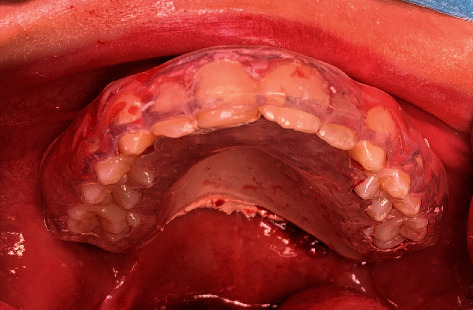
The wound was covered with a fabricated palatal acrylic plate.

**Figure 6:**
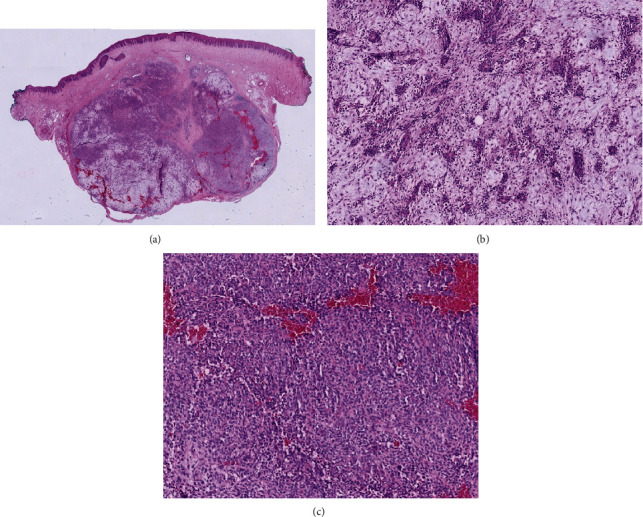
(a–c) Histopathological examination of the tissue showed a mucosal excision. In the submucosal stroma, there was a multinodular tumor consisting of 2 components. One component showed solid fields of cells with round to oval nuclei and eosinophilic cytoplasm. There was also a component of spindled cells within a myxoid matrix, with perivascular condensation of the cells. The tumor was clearly demarked from the surrounding stroma.

**Figure 7:**
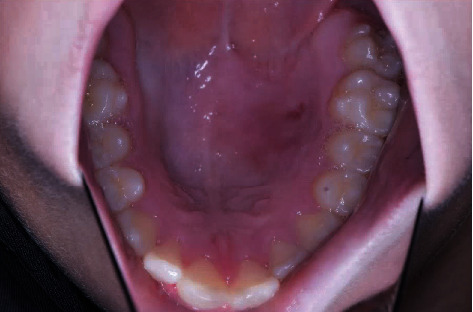
Photograph of the postoperative healing 4 months after surgery.

A healthy 12-year-old boy was referred by an orthodontist with swelling of the palate. The patient did not have any symptoms, and he had no pain or difficulty with speech or swallowing and no history of trauma, fever, fatigue, anorexia, respiratory or gastrointestinal symptoms, or weight changes. The swelling had been present for approximately 5 years, and the dentist observed it during the regular dental control visits, but no further action was taken. The swelling had never bothered the patient. The patient used Ritalin for attention deficit hyperactivity disorder (ADHD). Upon physical examination, a 2 cm nonfluctuating, nontender, immobile, firm nodular swelling was observed with intact overlying mucosa on the left side of the hard palate in the bicuspid-molar region (Figure 1). Clinical and radiographic examination of the dentition did not reveal any pathology. The patient had no palpable cervical lymphadenopathy. MRI showed a well-demarcated mass of the left side of the hard palate without bone invasion (Figures 2(a) and 2(b)). Pathological diagnosis was established by incisional biopsy performed under local anesthesia; histological examination was consistent with a pleomorphic adenoma of the minor salivary glands of the palate. The tumor was excised under general anesthesia with nasotracheal intubation. The mucosa around the tumor was marked and incised with an adequate margin, followed by dissection, with removal of the whole encapsulated mass and the mucoperiosteum (Figure 3). With a bur under copious sterile saline irrigation, the superficial part of the underlying bone was removed to ensure that no remnants of the lesions could cause recurrence. The defect on the palate was not reconstructed (Figure 4). An iodine tampon dressing was placed, and the wound covered with a fabricated palatal acrylic plate (Figure 5). The postoperative recovery was uneventful and histopathological assessment of the operation specimen confirmed the original diagnosis of pleomorphic adenoma of the palate. Examination showed mucosal tissue with a multinodular lesion consisting of two components in the stroma. There were solid fields of cells with round-oval nuclei and poorly delineated eosinophilic cytoplasm. There was also a spindle cell component located in the myxoid matrix with perivascular condensation. The tumor itself was sharply delineated (Figures 6(a)–6(c)). The wound gradually healed by secondary intention, and complete healing was observed 4 months after surgery (Figure 7). Follow-up 12 months after surgery did not reveal any recurrence. The patient remains under surveillance.

## 3. Discussion

Any swelling of the hard palate should be considered a possible minor salivary gland tumor. Pleomorphic adenoma is the most common benign salivary gland tumor but is mainly observed between 30 and 60 years of age [1]. This tumor is rarely observed in children, and involvement of the minor oral salivary gland is even less common. After the minor salivary glands, the palate is the most common intraoral location, followed by the upper lip and buccal mucosa [3, 4]. In a retrospective study of 4341 cases of pleomorphic adenoma, 90 patients younger than 18 years old were identified [1]. Among the 90 pediatric cases, the palatal minor glands were involved in 8 (8.9%).

Pleomorphic adenomas present as a painless, slow-growing firm mass, and patients generally do not exhibit symptoms. In our case, the swelling had been present for years and the patient did not have any symptoms. However, two other pediatric case reports noted that the swelling developed in 4 days and 2 weeks [5, 6].

A biopsy with histopathological assessment is mandatory to establish the diagnosis, especially as there seems to be a predisposition toward malignancy with pediatric salivary gland tumors [7, 8]. Salivary gland lesions are notorious for their extraordinary heterogeneity. However benign and low-grade neoplasms can often be accurately distinguished preoperatively from high-grade malignant tumors to guide clinical management [9]. Additional CT or MRI evaluation is useful in determining the size and extent of palatal lesions, as well as verifying destruction or erosion of the underlying bone [1].

The differential diagnosis of a firm palatal swelling of nonodontogenic origin in children, other than pleomorphic adenoma, is malignant salivary gland tumors, such as mucoepidermoid carcinoma, adenocarcinoma, or acinic cell carcinoma, which emphasizes the importance of histological assessment of swelling of the palate [10]. Other benign and malignant mesenchymal lesions that should be considered are neurofibroma and rhabdomyosarcoma, and lymphomas should be ruled out [11].

The optimal treatment for pleomorphic adenomas of the palate is a wide local surgical excision with an adequate margin of normal surrounding tissue. Complete surgical extirpation is critical to obviate the risk of future malignancy [12]. Simple enucleation should be avoided, as this can lead to local recurrence, particularly if the capsule breaks during surgery [13]. The underlying bone undergoes curettage with a sharp spoon or bur to avoid recurrence, and a local flap is used to reconstruct the palatal mucosal defect [2]. In the present case report, the palatal defect was not reconstructed, but a palatal prosthesis was used to cover the defect to allow it to heal by secondary intention. Recurrence of pleomorphic adenoma of the palate is seldomly seen but is a concern that makes long follow-up of patients necessary [11]. Recurrence on the palate can be considered serious, as they can enter the palatine foramen and reach the base of the skull [10].

## 4. Conclusion

We described a case of pleomorphic adenoma of the palate in a pediatric patient. The treatment was wide surgical excision of the pleomorphic adenoma with tumor-free margins. Primary reconstruction is not always necessary, as the defect can heal with secondary intention if the bony delimitation between the oral and nasal cavity remains intact.

## Data Availability

The data that support the findings of this study are available from the corresponding author upon reasonable request.
